# Case of Erysipelas Followed by General Anasarca, Succeeding the Vaccine Inoculation, and Terminating Fatally

**Published:** 1801-08

**Authors:** 


### *Case of Eryſipelas followed by general Anaſarca, ſucceeding the Vaccine Inoculation, and terminating fatally.*

Communicated by Mr. Clutterbuck.

SARAH WHITBURNE, reſiding at No. 12, Little James Street, Bedford Row, was inoculated with the Vaccine matter in the left arm, at the Small-pox Hoſpital, where ſhe was admitted an out-patient, on the 12th of May, 1801. She was ſeven weeks old at the time, was ſuckled by the mother, and in all reſpects a fine healthy child, except being occaſionally diſordered in her bowels. The parents were, to all appearance, in the beſt health.

On the 9th day, the arm having the uſual marks of infection, the child ſickened, had much fever, and was at times much convulſed. On the 10th, the inflammation ſurrounding the inoculated part was very intenſe, and the lymphatic glands ſituated on the left ſide of the neck were obſerved to be ſwollen with much redneſs in the ſkin covering them: there was, however, no continuity of inflammation from the arm to the neck, the former having the ordinary circumſcribed appearance. A copious raſh had at this time appeared over the whole body, but in two days after wholly vaniſhed. The arm and neck were directed to be fomented with flannels wrung out of hot water, with the effect of removing the inflammation from thoſe parts: the inoculated part ſpeedily dried and ſcabbed over, and did not afterwards ſhow any diſpoſition to inflame or ulcerate.

From the neck, however, the inflammation ſpread on to the face and head, which became much ſwelled, with the uſual appearances of eryſipelas on theſe parts, the eye-lids being nearly cloſed, and numerous veſications ariſing on the ſurface of the ſkin. The diſeaſe continued to creep along the cheſt, and gradually over the whole ſurface of the body, leaving the part before affected as it paſſed on to the neighbouring ſkin. The elbow and fore-arm on the inoculated ſide were ſo much ſwelled and inflamed, that ſuppuration ſeemed at one time to be threatened. It is curious that there was no return of inflammation on the inoculated part, or its immediate vicinity; the diſpoſition to eryſipelatous inflammation appearing to have been completely prevented by the original Vaccine inflammation.

A fortnight elapſed before the eryſipelas had completed its courſe, dating from its commencement on the 9th day after inoculation; and as the inflammation ſubſided, anaſarcous appearances ſucceeded, affecting eſpecially the extremities and *labia pudendi*, which were enormouſly ſwelled. Great general irritation, as might have been expected in so young a child, accompanied the disease, with frequent returns of convulsion, and at length insensibility; the hydropic effusion continuing to increase till the period of the child's death, which took place on the 15th of June, just five weeks after the inoculation.

The case which has now been described can be considered in the light of an exception only, and certainly affords no argument against the practice of the Vaccine inoculation; yet we can scarcely hesitate, I think, to refer the erysipelas, and consequently, *indirectly*, the death of the patient to this source. The commencement of the inflammation in the neck corresponding so exactly with the constitutional affection on the 9th day from inoculation, marks pretty clearly the relation of one to the other. The Vaccine poison, we know, is more ready to excite erysipelatous than phlegmonous inflammation; in which respect it differs from the Variolous. Although, therefore, the spreading and extent of the disease are probably to be referred to a constitutional cause, they were not so likely to have taken place under the Variolous as under the Vaccine Inoculation.

Whether the erysipelatous affection was owing to the absorption of the Vaccine poison, and the general action of this in the system, rather than to sympathy, admits, perhaps, of a question. The enlargement of the lymphatic glands on the side of the neck would seem to render such a supposition not improbable: yet it has not been demonstrated, I believe, that the absorbents pursue this route in their progress to the subclavian vein. This, however, is no argument against such a communication existing, as it is little more than the main trunks of these vessels that have been traced by dissection. The place where the puncture was made was situated a good deal higher than the insertion of the deltoid muscle, and considerably above the lymphatic trunks which pass up the arm in their progress to the axilla; and no branches are delineated in Mr. Cruikshank's *tables*, as taking this rout from the superior part of the humerus; absorbent vessels, therefore, may pass from the part in question to the lymphatic glands of the neck, although they have not been traced by the knife of the anatomist.

Two cases very similar to the above are given by Mr. Maddock, of Nottingham, in the 24th Number of the Med. and Phys. Journal, one of which terminated fatally on the 26th day after inoculation; the other patient recovered after five weeks duration of the erysipelas, an abscess, apparently critical, taking place in the axilla. The unfavourable cases related in the 22d Number of the same Journal, as occurring in the neighbourhood of Clapham, and which (unfairly I think) were attempted to be ascribed to some negligence on the part of the practitioner, tioner, were probably of the fame nature with thofe above mentioned. In the inftance here related, no doubt can be entertained of every neceffary circumftance having been attended to in the operation.

It may be proper to obferve, that no prejudice is likely to arife againft the Vaccine Inoculation by the fatal termination of Whitburne's Cafe; the mother fuppofing the child to have been inoculated for the Small-pox, and attributing its death to the difappearance of the eruption, or, as fhe terms it, the ftriking-in of the pock on the 10th day. The only inference the cafe feems to furnifh, is, that we fhould not under-rate the *poffible* danger of the difeafe, and thereby afford a handle to prejudice or malevolence to oppofe a moft ufeful and important practice.

P. S. It is almoft fuperfluous now to adduce evidence in favour of the new Inoculation; and I agree with Dr. Pearfon, in thinking it beft to bring forward unfavourable cafes only, when fuch occur. I may obferve, however, that I have met with two inftances lately of the *permanently* preventive power of the Vaccine. Two female fervants of Mr. Cater, of Gracechurch Street, were inoculated with Vaccine matter, the one two years and a half, the other fifteen months ago. They have both been employed lately in nurfing a child of Mr. C's in Small-pox, with a numerous crop of puftules; and neither of them have fuffered.

*Walbrook, July* 12, 1801.

